# DARPin_9-29-Targeted Gold Nanorods Selectively Suppress HER2-Positive Tumor Growth in Mice

**DOI:** 10.3390/cancers13205235

**Published:** 2021-10-19

**Authors:** Galina M. Proshkina, Elena I. Shramova, Marya V. Shilova, Ivan V. Zelepukin, Victoria O. Shipunova, Anastasia V. Ryabova, Sergey M. Deyev, Alexander B. Kotlyar

**Affiliations:** 1 Shemyakin-Ovchinnikov Institute of Bioorganic Chemistry, Russian Academy of Sciences, Miklukho-Maklaya St, 16/10, 117997 Moscow, Russia; gmb@ibch.ru (G.M.P.); fei@ibch.ru (E.I.S.); mari_shilova29@mail.ru (M.V.S.); zelepukin@phystech.edu (I.V.Z.); vika_shipunova@mail.ru (V.O.S.); 2 MEPhI (Moscow Engineering Physics Institute), Institute of Engineering Physics for Biomedicine (PhysBio), 31 Kashirskoe Shosse, 115409 Moscow, Russia; 3 Prokhorov General Physics Institute of the Russian Academy of Sciences, 38 Vavilova St, 119991 Moscow, Russia; nastya.ryabova@nsc.gpi.ru; 4 Department of Biochemistry and Molecular Biology, George S. Wise Faculty of Life Sciences and the Center of Nanoscience and Nanotechnology, Tel Aviv University, Ramat Aviv, Tel Aviv 69978, Israel

**Keywords:** cancer, DARPin, gold nanorods, mice treatment, near-infrared illumination, photothermal therapy

## Abstract

**Simple Summary:**

Breast cancer is one of the main causes of cancer-related death in women all around the world. The disease becomes largely incurable and lethal after metastasis to distant organs. High level of HER2, a tyrosine kinase receptor, is associated with more aggressive clinical behavior and poor prognosis for breast cancer patients. In this paper, we developed a novel nano-biomaterial for selective photothermal therapy of HER2-positive breast cancers. We demonstrate that bovine serum albumin (BSA)-coated mini gold nanorods (GNRs) chemically conjugated with a HER2-specific designed ankyrin repeat protein, DARPin_9-29, selectively accumulate in HER2-positive xenograft tumors in mice and lead to a strong reduction in the tumor size when being illuminated with near-infrared light.

**Abstract:**

Near-infrared phototherapy has great therapeutic potential for cancer treatment. However, for efficient application, in vivo photothermal agents should demonstrate excellent stability in blood and targeted delivery to pathological tissue. Here, we demonstrated that stable bovine serum albumin-coated gold mini nanorods conjugated to a HER2-specific designed ankyrin repeat protein, DARPin_9-29, selectively accumulate in HER2-positive xenograft tumors in mice and lead to a strong reduction in the tumor size when being illuminated with near-infrared light. The results pave the way for the development of novel DARPin-based targeted photothermal therapy of cancer.

## 1. Introduction

Breast cancer is one of the main causes of cancer-related death in women all around the world [1,2]. The disease can be treated at early stages but is almost incurable when metastasis has occurred. A high level of human epidermal growth factor receptor 2 (HER2) is associated with the aggressive development of breast cancer [3,4,5,6]. Trastuzumab, a HER2-specific monoclonal antibody, has been used in clinical practice to treat HER2-positive breast cancer for about 20 years [7,8,9,10,11]. Designed ankyrin repeat proteins (DARPins), which are characterized by high-affinity interaction with different epitopes of HER2, have also been shown to specifically target HER2-positive cells [12,13,14,15,16,17,18]. Conjugates of DARPins with gold nanostructures have been specifically delivered to HER2-positive cells and the conjugate-targeted cells selectively eradicated by near-infrared (NIR) photothermal therapy [19,20]. We recently demonstrated [20] that DARPin_9-29-coated gold nanorods (GNRs), DARPin-GNRs, efficiently suppress cancer cells’ growth in vitro. However, despite multiple attempts, there was no noticeable effect of the conjugate on tumor growth in animals. The reason for that was that the DARPin-coated nanorods aggregated in blood vessels after the injection.

To overcome the challenge of low colloidal stability of the conjugate, here we report the synthesis of novel highly stable DARPin-functionalized nanorods. In contrast to earlier reported DARPin-GNRs, in which DARPin_9-29 molecules were attached directly to the GNR surface, the nanorods here were first coated with bovine serum albumin (BSA) and then conjugated with the DARPin. It has been shown [21,22,23] that coating with the protein strongly increases the colloidal stability and biocompatibility of gold nanostructures. The DARPin was attached to the BSA coating layer here using a heterobifunctional crosslinker (sulfo-EMCS) that covalently conjugates amine- and sulfhydryl-containing molecules. The resulting conjugate, DARPin-BSA-GNR, is much more stable than DARPin-GNR and does not aggregate at very high (1M) salt concentrations. DARPin-BSA-GNR was shown to bind to HER2-positive cells not less tightly and specifically than DARPin-GNR. In contrast to the latter conjugate, DARPin-BSA-GNRs did not aggregate in blood and specifically accumulated in HER2-positive tumors. Moreover, NIR illumination of the tumor area in DARPin-BSA-GNR-treated mice significantly suppressed the primary and metastatic tumor growth.

## 2. Materials and Methods

Unless otherwise noted, reagents and chemicals were purchased from Sigma-Aldrich (St. Louis, MO, USA).

### 2.1. Cell Cultures

BT-474 cells (human ductal carcinoma, ATCC HTB-20), BT-474 cells stably expressing NanoLuc [24] and MDA-MB-231 cells (mammary gland/breast adenocarcinoma, ATCC HTB-26) were cultured in RPMI-1640 culture medium supplemented with 10% fetal bovine serum (HyClone), 10 U/mL penicillin (PanEco), 10 μg/mL streptomycin (PanEco) and 2 mM *L*-glutamine (PanEco). Cells were grown in a humidified atmosphere with 5% CO_2_ at 37 °C.

### 2.2. Preparation of DARPin_9-29

DARPin_9-29 was produced in *Escherichia coli.* For this purpose, the plasmid pET22-DARP_9-29-encoded *DARPin* gene was transformed in the bacterial strain BL21(DE3) (Novagen-EMD Millipore, Madison, WI, USA). For protein production, the auto-induction method was used [25]. In short, cells were grown at 25 °C for 16 h in ZYM-5052 medium [25] supplemented with 100 μg/mL ampicillin. After this stage, all manipulations were carried out at 4 °C. Cells were pelleted on a Thermo Jouan KR25 centrifuge at 5000× *g* for 15 min and resuspended in 20 mM NaPi (pH 7.5), 0.5 M NaCl, 1 mM PMSF and 55 μg/mL lysozyme. Cell walls were destroyed using a Vibra Cell ultrasonic liquid processor VCX130 (Sonics & Materials, Inc., Newtown, CT, USA). Then, cellular debris was pelleted at 25,000× *g* for 1 h, and the supernatant was mixed with imidazole (to 30 mM in final solution), filtered (the size of the membrane pore was 0.22 μm) and loaded onto a HisTrap HP column (1 mL, GE Healthcare, Chicago, IL, USA). Protein purification was carried out according to the manufacturer’s instructions. The purity of the protein was confirmed by 12% SDS-PAGE, and the concentration was determined spectrophotometrically using an extinction coefficient ε_275_ equal to 4.65 mM^−1^ cm^−1^.

### 2.3. Synthesis of Mini Gold Nanorods (GNRs)

For gold nanorods (GNRs) preparation, the seed-mediated growth method was used [20,26]. In short, a seeds solution was prepared as follows: 25 μL of 10 mM AuHCl_4_ was added to 1 mL of 0.1M CTAB. Two minutes later, 60 μL of 10 mM NaBH_4_ was added to the stirred solution. After vigorous stirring for 10 min, the solution was left unstirred for 1.5 h. Then, 9 mL of 0.1M CTAB, 0.5 mL of 10 mM AuHCl_4_, 125–150 μL of 10 mM AgNO_3_, 200 μL of 1M HCl and 80 μL of 0.1M ascorbic acid were successively added to a 20-milliliter scintillation vial under gentle stirring in sequence. After the solution became colorless (about 1 min), 1 mL of the seeds was mixed into the solution and gently stirred. The solution was placed in the dark overnight at RT. The next day, the solution was placed in a 15-milliliter Corning tube on ice until CTAB crystals were formed. The tube was centrifuged for 4 min at 1000 rpm, 4 °C (Eppendorf 5810R); the supernatant was moved to a new tube. This process was repeated twice. The supernatant after the last cooling/centrifugation cycle was heated for 10 min at 40 °C and centrifuged at 25 °C for 4 min at 8000 rpm (Eppendorf 5418). The supernatant was then centrifuged for 10 min at 14,000 rpm (Eppendorf 5418). The pellets were pooled and resuspended in ~1.5 mL of deionized water. The pellets were pooled; the final volume was 50 μL. The dimensions of the resulting GNRs were about 7–8 nm in diameter and ~50 nm in length.

### 2.4. Coating of GNRs with BSA and Conjugation with DARPin_9-29

**Figure 1:**
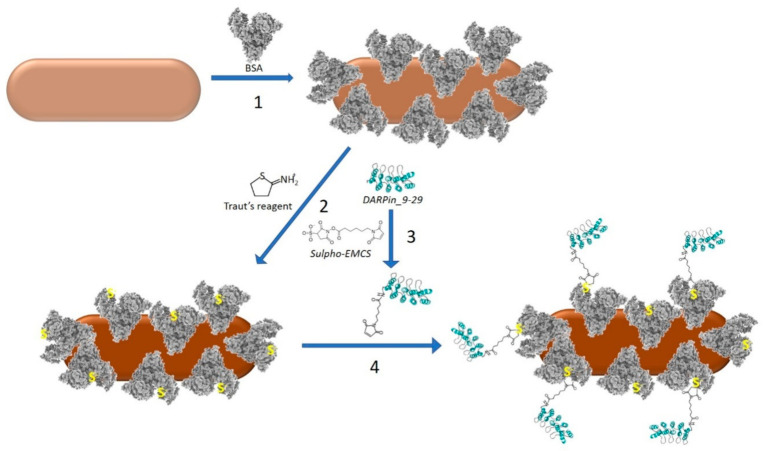
Scheme of DARPin-BSA-GNR synthesis. 1: Coating of a GNR (light brown) with BSA (grey) molecules. 2: Modification of primary amino groups of BSA molecules in the coating layer by Traut’s reagent. This reaction introduces SH groups (shown in yellow) into proteins. 3: Attachment of sulfo-EMCS to one of the primary amino groups of the DARPin (shown in aqua). 4: Covalent attachment of the sulpho-EMCS-modified DARPin molecules to SH groups of the Traut-reagent-treated BSA-coated GNR.

Preparation of the DARPin-BSA-GNRs included the following steps (Figure 1).

#### 2.4.1. Coating of GNRs with BSA

The concentrated nanorods (see Section 2.3 and Figure 1, step 1) were mixed into 1 mL of 100 mg/mL BSA solution and incubated for 16 h under ambient temperature. The nanorods were centrifuged for 30 min at 14,000 rpm on a table centrifuge, resuspended in 0.5 mL of 100 mM K-Pi (pH 7.5) and passed through a Sepharose CL-2B column (15 × 80 mm) equilibrated with 100 mM K-Pi (pH 7.5). The fraction containing BSA-coated GNRs (BSA-GNRs) was collected.

#### 2.4.2. Modification of BSA-GNRs with Traut’s Reagent 

The BSA-GNRs obtained in Section 2.4.1 were incubated with 6 mM 2-iminothiolane hydrochloride (Traut’s reagent, Sigma, St. Louis, MO, USA) at 25 °C for 45 min (Figure 1, step 2). To separate BSA-GNRs from the unbound Traut’s reagent, the reaction mixture was passed through a desalting column (NAP-10, GE Healthcare). Elution was performed in 20 mM K-Pi (pH 7.5). BSA-GNRs conjugated with Traut’s reagent were collected in the void volume fraction.

#### 2.4.3. Modification of DARPin_9-29 with sulfo-EMCS 

DARPin_9-29 (1 mL; 6 mg/mL) was incubated with 1.5 mM sulfo-EMCS for 45 min under ambient temperature (Figure 1, step 3). The reaction mixture was applied on an NAP-10 desalting column (GE Healthcare). DARPin_9-29 conjugated with sulfo-EMCS was eluted in the void volume fraction.

#### 2.4.4. Conjugation of DARPin_9-29 with BSA-GNRs 

An amount of 0.5 mL of the sulfo-EMCS-treated DARPin (Section 2.4.3, Figure 1, step 4) was mixed with an equal volume of the Traut-treated BSA-GNRs (Section 2.4.2) and incubated for 1 h at RT. The DARPin and the linker were separated from BSA-GNRs on a Sepharose CL-2B column (10 × 35 mm) equilibrated with 20 mM K-Pi (pH 7.5). The void volume fraction containing DARPin-BSA-GNRs was collected and stored at 4 °C.

### 2.5. Confocal Microscopy

BT-474 and MDA-MB-231 cells were cultured on glass-bottomed dishes (WillCo Well, Amsterdam, The Netherlands) overnight in 5% CO_2_ at 37 °C. DARPin-BSA-GNRs (250 nM) labeled with fluorescein were added to the cells and incubated for 10 min at 37 °C. The dye-labeled DARPin-BSA-GNRs were prepared as follows: the conjugate (250 nM; 1 mL) was incubated with 1.25 μM fluorescein isothiocyanate for 1 h in 20 mM K-Pi (pH 8.0). To separate the DARPin-BSA-GNRs-FITC from unbound dye, an NAP-25 desalting column (GE Healthcare) equilibrated with 20 mM K-Pi (pH 7.5) was used. To be visible in confocal microscopy, the nuclei were stained with 1 µM Hoechst 33342 (Invitrogen, Waltham, MA, USA) for 10 min at 37 °C. The imaging was performed with a laser scanning microscope (Carl Zeiss LSM-980, Jena, Germany). Fluorescein was excited at 488 nm; the emission was detected between 500 and 550 nm. Hoechst was excited at 405 nm and the emission was detected in the 410–490-nanometer range. A 63× oil Plan-Apochromat objective with a numerical aperture of 1.4 was used.

### 2.6. MTT Assay

The phototoxicity of the nanorods was determined using the MTT assay [27]. BT-474 or MDA-MB-231 cells were seeded on a 96-well plate (10^4^ cells per well) in 100 μL of RPMI-1640 complete medium and were cultured overnight. Then, DARPin-BSA-GNRs or BSA-GNRs were added to the wells, incubated for 10 min at 37 °C, washed three times with cell culture medium (RPMI-1640) and illuminated at 850 nm for 20 min using an infrared LED light source (30 mW/cm^2^, Elixa, Chicago, IL, USA). Fresh medium was added to the wells after the illumination and the cells were incubated for 72 h at 37 °C. Then, the medium was replaced with 0.5 g/L MTT (3-(4,5-dimethylthiazol-2-yl)-2,5-diphenyltetrazolium bromide) solution. Incubation was carried out for 1 h at 37 °C. During this time, mitochondrial dehydrogenases reduced MTT to formazan, which was then dissolved in DMSO (100 μL per well). After complete dissolving of formazan, the absorbance was measured spectrophotometrically at 570 nm with an Infinite 1000 Pro reader (Tecan, Grödig, Austria).

### 2.7. Flow Cytometry

To determine DARPin-BSA-GNRs’ ability to recognize the HER2 receptor, HER2-positive BT-474 cells were detached from the substrate, washed with PBS, resuspended in 500 µL of complete growth medium at a concentration of 10^6^ cells per mL and incubated with 200 nM DARPin-BSA-GNR-FITC-labeled conjugate and 250 or 25 nM free DARPin for competition. Incubation was carried out for 10 min at 37 °C. Samples were washed 3 times with PBS and analyzed using a Novocyte 3000 VYB flow cytometer (ACEA Biosciences, San Diego, CA, USA) using a 488-nanometer excitation laser and a 530/30-nanometer emission filter.

### 2.8. Laboratory Animals

Laboratory animals (female immunocompromised mice, aged 6 to 8 weeks) were obtained from the licensed SPF (specified pathogen-free) nursery of the Shemyakin–Ovchinnikov Institute of Bioorganic Chemistry (Russian Academy of Sciences). Mice were kept in specific-pathogen-free facilities with free access to food and water. All experimental procedures were approved by the Animal Care and Use Committee of the Shemyakin–Ovchinnikov Institute of Bioorganic Chemistry, protocol No. 298/2020, dated 29 May 2020.

Mice were inoculated subcutaneously in the right flank with 2 × 10^6^ BT-474 or BT/NanoLuc cells per mouse in 30% Matrigel (Corning, Glendale, AZ, USA). The greatest transverse diameter (width) and the greatest longitudinal diameter (length) of a tumor were determined with a Vernier mechanical caliper. The tumor volume (V) was calculated using the following formula: V = length × width^2^/2 [28]. Biodistribution studies or therapy was started when the average tumor size was about 90 mm^3^ (10–12 days after inoculation).

### 2.9. Biodistribution of GNRs in Tumor-Bearing Mice

To female BALB/c nude mice xenografted subcutaneously with HER2-positive BT-474 cells, 100 µL of BSA-GNR or DARPin-BSA-GNR suspensions in PBS containing 2% (*w*/*v*) BSA was intravenously injected once a day for 10 days. A group of animals (*n* = 3) was euthanized on day 10, and the organs were collected. The distribution of the conjugate in different organs and tumors was measured using ICP-MS. A slice of each organ or tumor was completely dissolved for 2–3 h in 80 μL of concentrated nitric acid. The solution was diluted 10 times with distilled water and centrifuged at 1000× *g* for 5 min. The Au content in the supernatant was measured with a NexION 2000 (Perkin Elmer, Boston, MA, USA) mass spectrometer. The device was calibrated using standard aqueous AuHCl_4_ solutions. 

### 2.10. Therapy

All experimental procedures were approved by the Animal Care and Use Committee of the Shemyakin–Ovchinnikov Institute of Bioorganic Chemistry, protocol No. 298/2020, dated 29 May 2020. Female BALB/c nude mice were inoculated subcutaneously in the right flank with 2 × 10^6^ BT-474/NanoLuc cells in 30% Corning Matrigel per mouse. Ten days later, when the average tumor volume reached 90 ± 31 mm^3^, the mice were randomized into groups (*n* = 4 per group). An amount of 100 μL of 30 nM nanorod (DARPin-BSA-GNR or DARP-GNR) suspension in PBS was intravenously injected every other day for 10 days. The tumor area in the mouse body was illuminated with a 120-LED array of 850 nm (30 mW/cm^2^, Elixa) every day for 20 min; all 120 LEDs were turned on. The control group was injected with PBS only; the illumination was as for the first group. The tumor volumes were monitored as described above along with body weight. Mice were euthanized when the subcutaneous tumor reached a volume of ~1000 mm^3^. The tumor growth inhibition (TGI) was calculated using the following formula: TGI (%) = [(V_control_ − V_treatment_) × 100%]/V_control_, where V is the tumor volume at a certain time point.

On the first and last days of the treatment, BT-474/NanoLuc tumor-bearing mice were imaged with an IVIS Spectrum CT system (Perkin Elmer, Boston, MA, USA). During the bioimaging process, mice were anesthetized with isoflurane. Highly specific NanoLuc substrate furimazine administered i.p. (7 μg per mouse) was used to induce luminescence in animals; data acquisition was started one minute after the furimazine administration. All bioluminescence data have been normalized to the acquisition conditions and are displayed in radiance (photons/s/cm^2^/str).

## 3. Results and Discussion

As we reported earlier [20], conjugates of mini gold nanorods (7−8 nm in diameter and ∼50 nm in length) with DARPin_9-29 molecules specifically bind to HER2-positive cells. Illumination with NIR light led to strong eradication of the conjugate-treated cells. Although highly potent in vitro, the conjugate was inefficient at suppressing tumor growth in tumor-bearing mice. Injection of the conjugate did not lead to accumulation of gold in the tumors of BT-474/NanoLuc tumor-bearing mice (Table 1).

No significant PTT-induced inhibition of tumor growth in the DARP-GNR-treated mice was observed (Figure S1). We noticed that soon after the administration of the conjugate into the mouse tail vein, the injection area turned blue. This indicates the relatively low stability of the conjugate under physiological conditions and, therefore, its aggregation in the animal’s blood vessels. To surmount this challenge, we have developed a method for the preparation of a novel highly stable GNR construct. The method consists of two main steps: firstly, coating of the nanorod with BSA molecules, and secondly, covalent conjugation of BSA-GNR conjugates with DARPin_9-29 to yield the final construct, DARPin-BSA-GNR (Figure 1).

**Figure 2:**
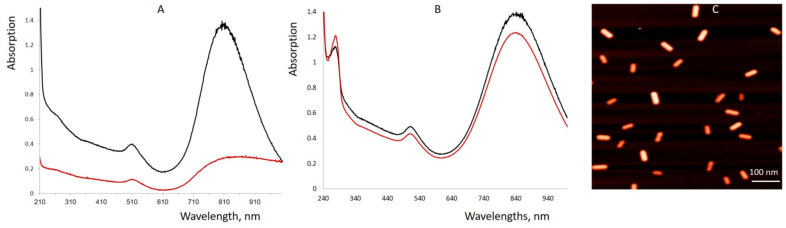
Absorption spectra (**A**,**B**) and AFM image (**C**) of DARPin-GNRs (**A**) and DARPin-BSA-GNRs (**B**,**C**). (**A**,**B**): The nanorods were incubated for 1 h at 37 °C in water (black curves) and in 1 M NaCl (red curves). (**C**): AFM was performed on molecules adsorbed on muscovite mica as described in [20].

DARPin_9-29 was attached to the BSA layer of the nanorod using a heterobifunctional crosslinker (sulfo-EMCS). The linker was covalently attached to one of the amino groups of the DARPin through a succinimide moiety (Figure 1; reaction 3). The maleimide moiety on the opposite end of the linker was reacted with one of the SH groups of the BSA coating layer. The thiols were introduced into the protein layer by treatment of BSA-GNRs with Traut’s reagent (Figure 1; reaction 2). As seen in Figure 2C and Figure S2, DARPin-BSA-GNRs are rather uniform. The DARPin-BSA-GNRs move as a single band through the gel in the electrical field, while the non-coated nanorods aggregate in the running buffer. This, along with the AFM data (Figure 2C), shows that DARPin-BSA-GNRs are rather uniform and stable. The novel conjugate (DARPin-BSA-GNR), in contrast to the earlier reported one (DARPin-GNR), did not aggregate even in 1M NaCl. Only a slight decrease in absorption was observed after 1-h incubation of DARPin-BSA-GNRs in the presence of salt; DARPin-GNRs were almost completely bleached during the incubation (Figure 2). This demonstrates that coating with BSA strongly increases the colloidal stability of the nanorods. 

**Figure 3:**
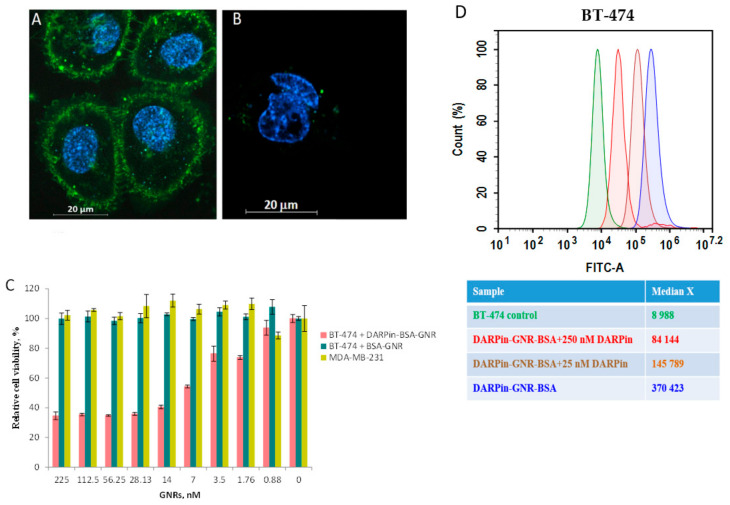
In vitro characterization of DARPin-BSA-GNRs. Confocal images of HER2-overexpressing BT-474 cells (**A**) and MDA-MB-231 cells (**B**) characterized by HER2 expression normal for epithelial cells. Fluorescence-merged images of cells in green (FITC-labeled DARPin-GNRs) and blue (Hoechst 33342) channels are presented. (**C**) In vitro cell viability of BT-474 cells treated with DARPin-BSA-GNRs (pink columns) or BSA-GNRs (cyan columns) and MDA-MB-231 cells treated with DARPin-BSA-GNRs (dark yellow columns) and exposed to illumination. Error bars represent the standard deviation (*n* = 3). (**D**) Normalized flow cytometry histograms showing specific interaction of DARPin-BSA-GNRs with HER2 receptors on the surface of BT-474 cells in the absence (blue peak) and presence of 250 (red peak) or 25 nM (brown peak) free DARPin.

The ability of DARPin-BSA-GNR conjugates to specifically interact with HER2 was checked in vitro. For this, HER2-positive BT-474 cells expressing 10^6^ receptors per cell [29] and MDA-MB-231 cells expressing a normal epithelial cell receptor amount (10^4^ receptors per cell) [30] were used. We demonstrated that DARPin-BSA-GNRs bind specifically to the surface of HER2-positive cells (Figure 3A) and only slightly stain the membrane surface of MDA-MB-231 cells (Figure 3B). Similar to DARPin-GNR [15], illumination by NIR light led to the strong eradication of only DARPin-BSA-GNR-treated HER2-positive BT-474 cells (Figure 3C). The IC_50_ calculated using nonlinear regression was equal to 4.8 nM. BT-474 cells treated with non-targeted BSA-GNRs did not reveal any signs of death under illumination (Figure 3C, cyan column). The viability of MDA-MB-231 cells characterized by normal HER2 expression was almost not affected by the illumination as well (Figure 3C, dark yellow column).

To confirm that the binding of DARPin-BSA-GNRs to cells occurs through interaction of the DARPin moieties with HER2 receptors on the cell surface, the competition test was used. Free DARPin was used as a competitive agent. The results of the flow cytometry measurements indicate that the HER2 recognition property of DARPin-BSA-GNRs correlates with the free DARPin concentration in the mixture: BT-474 cells treated with DARPin-BSA-GNRs revealed a significant (~41-fold) fluorescence intensity shift compared to untreated cells (Figure 3D, blue peak); cells treated with DARPin-BSA-GNRs and 250 or 25 nM DARPin revealed 9-fold (Figure 3D, red peak) and 16-fold (Figure 3D, brown peak) fluorescence intensity shifts, respectively. This clearly demonstrates that the interaction of DARPin-BSA-GNRs is HER2-specific.

In vivo studies were carried out using BT-474/NanoLuc [24] tumor-bearing mice. After the average tumor volume reached approximately 90 mm^3^, the mice were injected intravenously with 100 μL of 30 nM DARPin-BSA-GNRs or BSA-GNRs. The distribution of GNRs (gold) in main organs and tumors was measured 20 h after the injection using inductively coupled plasma mass spectrometry (ICP-MS). As seen in Table 1, the gold content in all tested organs was similar in mice treated with DARPin-BSA-GNRs and BSA-GNRs, while the gold content in the tumors of DARPin-BSA-GNR-treated mice greatly exceeded that estimated for BSA-GNR-treated ones. This clearly demonstrates that functionalization with DARPin_9-29 is essential for the accumulation of the nanorods in HER2-positive tumors.

**Figure 4:**
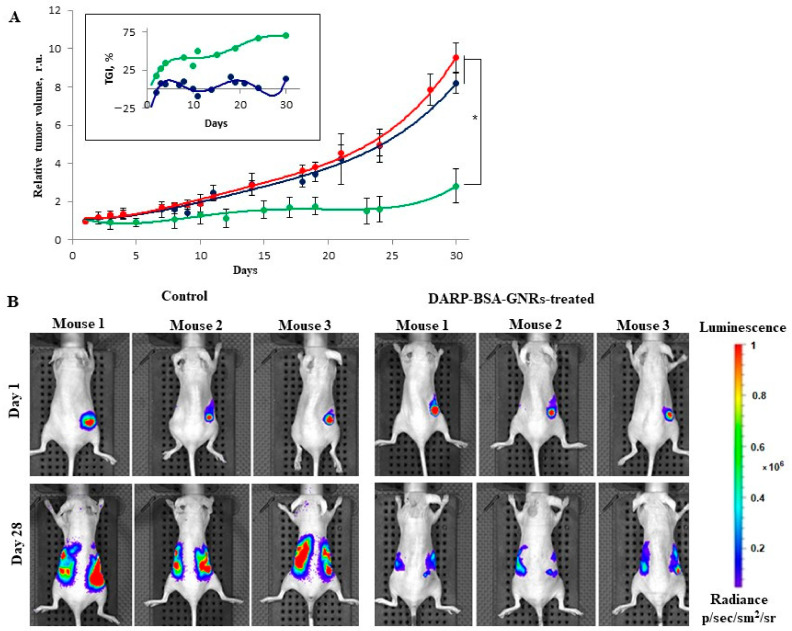
Effect of DARPin-BSA-GNRs on tumor growth dynamics in mice. (**A**) Mice were injected with PBS (red and blue curves) and DARPin-BSA-GNRs (green curve). The tumor areas in mice were illuminated by an NIR LED light source (blue and green curves). Bars indicate SD; * *p* < 0.05. Inset presents TGI value at every time point, calculated as described in the Materials and Methods section. (**B**) Imaging of BT474/NanoLuc tumor xenografts. The mice were injected with DARPin-BSA-GNRs (right panel) and PBS (left panel), illuminated by NIR light and imaged with an IVIS Spectrum CT system on the first (upper panels) and twenty-eighth (lower panels) days.

The dynamics of tumor growth were monitored (Figure 4). Eight BT-474/NanoLuc tumor-bearing mice with a tumor size of approximately 90 mm^3^ were randomly divided into two groups. Mice of the first group were injected intravenously with 100 μL of 30 nM DARPin-BSA-GNRs every other day, and the tumor area was illuminated with an 850-nanometer laser (30 mW/cm^2^) for 20 min every day; the treatment lasted for 10 days. Mice of the second (control) group were injected with 100 μL PBS and illuminated as the mice of the first group were. No significant changes in the body weight of the animals of both groups were observed during the treatment. The tumor volume in the mice of the control group increased by about nine times at day 32, while the tumor volume in the mice of the DARPin-BSA-GNR-treated group increased by only approximately three times (Figure 4A). Tumor growth inhibition (TGI, in %) for the treated groups was calculated for each time point (see insert to Figure 4A). It was about 70% in the DARPin-BSA-GNR-treated mice. The tumor growth was also monitored using an IVIS Spectrum CT system (Figure 4B). As can be seen in Figure 4B, the primary tumor and metastatic nodes grew in the mice of the control group much faster as compared to those in the animals of treatment group.

## 4. Conclusions

We previously demonstrated [20] that DARPin-GNRs bind specifically to HER2-overexpressing cells, and that illumination with NIR light (850 nm) greatly lowers the viability of the conjugate-treated cells. Unfortunately, despite our best efforts, we were unable to demonstrate the effect of DARPin-GNRs in vivo (Figure S1). To overcome this challenge, we synthesized novel highly stable DARPin-functionalized BSA-coated GNRs (DARPin-BSA-GNRs), which, in contrast to DARP-GNRs, did not aggregate after being injected into mice intravenously. In contrast to DARP-GNRs, the novel nanorod-DARPin conjugate was shown to specifically accumulate in the tumor (see Table 1). Illumination of the tumor area with an 850-nanometer laser (30 mW/cm^2^) led to about 70% inhibition of tumor growth in the conjugate-treated mice (Figure 4A). The strong and specific NIR light-induced suppression of tumor growth in mice demonstrated here can pave the way for application of the conjugate in PTT of cancer.

## Figures and Tables

**Table 1:** Distribution of gold in organs of tumor-bearing mice.

	Content of Gold in Organs and Tumor, %		
	DARPin-BSA-GNR-Treated Mice	BSA-GNR-Treated Mice	DARPin-GNR-Treated Mice
Liver	88.62 ± 2.00	95.51 ± 1.52	82.38 ± 7.13
Kidney	0.09 ± 0.02	0.09 ± 0.04	0.28 ± 0.02
Lungs	0.07 ± 0.01	0.23 ± 0.07	0.44 ± 0.24
Heart	0.02 ± 0.01	<0.01	0.03 ± 0.01
Spleen	7.67 ± 1.26	4.14 ± 1.41	16.90 ± 1.24
Tumor	3.44 ± 0.15	<0.01	0.22 ± 0.09

## Data Availability

All data are contained within the article and supporting information.

## Supplementary Materials

The following are available online at https://www.mdpi.com/article/10.3390/cancers13205235/s1. Figure S1: Tumor growth dynamics in DARPin-GNR-treated mice; Figure S2: Agarose gel electrophoresis of BSA-DARPin-GNRs.

## Abbreviations

DARPins, designed ankyrin repeat proteins; HER2, human epidermal growth factor receptor 2; GNRs, gold mini nanorods; PTT, photothermal therapy; NIR, near-infrared; BSA, bovine serum albumin.
