# Supplementary material for: DARPin_9-29-Targeted Gold Nanorods Selectively Suppress HER2-Positive Tumor Growth in Mice
Source: Cancers (Basel). 2021 Oct 19;13(20):5235. doi: 10.3390/cancers13205235 (PMC8534065; doi:10.3390/cancers13205235)
Supplement: Supplementary file 1 [file cancers-13-05235-s001.zip › cancers-1380696-supplementary.pdf]

cancers

Supplementary Materials

DARPin_9-29-Targeted Gold Nanorods Selectively Suppress HER2-Positive Tumor Growth in Mice

Galina M. Proshkina, Elena I. Shramova, Marya V. Shilova, Ivan V. Zelepukin, Victoria O. Shipunova, Anastasia V. Ryabova, Sergey M. Deyev and Alexander B. Kotlyar

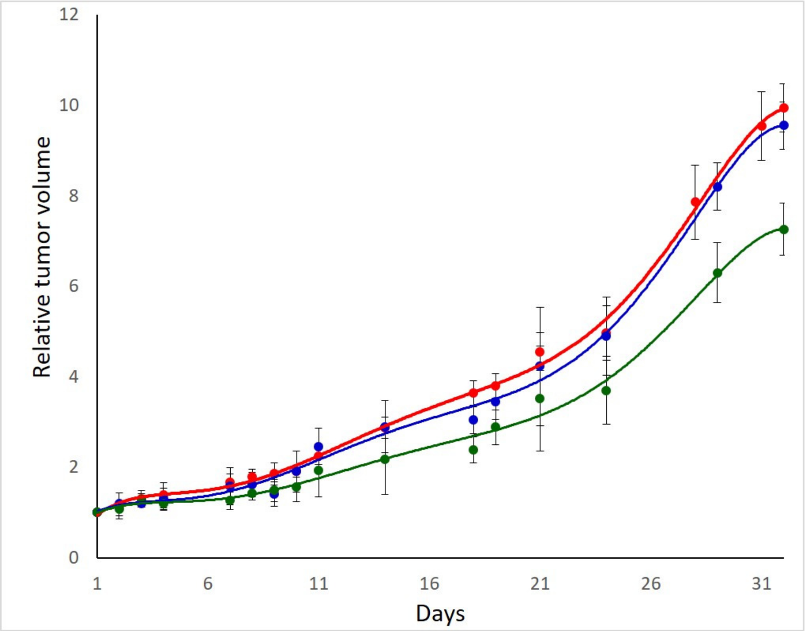

Figure S1. Effect of DARP-GNRs on tumor growth dynamics in mice. Mice were injected with PBS (red and blue curves) and DARP-GNRs (green curve). The tumour area in mice was illuminated by NIR laser (blue and green curves) as described in Materials and Methods. Bars indicate SD; $p < 0.05$.

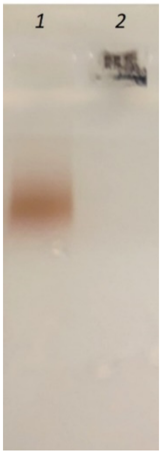

Figure S2. Electrophoresis of BSA-DARPin-GNRs (lane 1) and -bare GNRs (lane 2). The electrophoresis was conducted in 2% agarose gel as described in Materials and Methods.
